# Genu Recurvatum Following Paediatric Femoral Diaphyseal - Fracture : Salter Type V Injury Revisit

**DOI:** 10.5704/MOJ.1311.006

**Published:** 2013-11

**Authors:** A Cogan, ST Donell

**Affiliations:** Department of Orthopaedics and Trauma, Norfolk and Norwich University Hospital, Norwich, United Kingdom; Department of Orthopaedics and Trauma, Norfolk and Norwich University Hospital, Norwich, United Kingdom

## Abstract

A 17-year old boy with a history of a right femoral shaft fracture, fixed with a reamed intramedullary nail four years earlier, presented with a 15° genu recurvatum deformity, presumably due to premature closure of the anterior proximal tibial physeal plate following a Salter type V injury. He was treated with a supra-tubercular anterior opening wedge osteotomy, fixed with two Puddu plates and grafted with bone matrix substitute. The patient went on to unite without complication, but came back to clinic six years later with anterior knee pain and patella infera. The paper discusses genu recurvatum after growth plate arrest and the various techniques to address the problem. Moving the tibial tubercle by including it in the osteotomy should be considered to avoid the complication of patella infera.

## Introduction

Paediatric femoral shaft fractures are known to produce growth arrest at the proximal tibia, probably caused by compression injuries of the proximal tibial physis (Salter type V injuries), most often in the area of the tibial tubercle, producing late onset genu recurvatum deformities. The deformity is often severe enough to warrant operative correction, with surgery having its own set of long term complications, particularly anterior knee pain.

## Clinical History

In August 1999, a 17-year old boy sustained a closed unstable midshaft fracture of the right femur after falling off a tractor. He had a history of compulsive eating and was overweight (64 kg for 165cm - BMI 26.6). He was treated with a reamed intramedullary nail despite his age because his excessive weight made both parents and surgeons feel that external fixation or flexible nailing was inappropriate. The follow-up was uncomplicated and the fracture healed.

The intramedullary nail was removed in 2002 due to discomfort in the lower thigh, after which all symptoms were relieved. He was discharged from follow-up since the growth plates had closed. No leg length discrepancy was recorded.

**Fig. 1:**
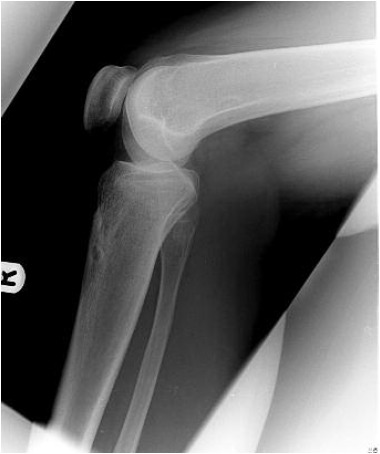
: Lateral Xray of the knee taken on 26/2/2003 showing anterior tibial slope.

He returned to the clinic in 2003 because of pain in his right knee coupled with hyperextension. The clinical examination showed a recurvatum of 15º, no effusion, intact ligaments and a full range of knee motion. His plain radiographs (Fig 1) revealed a 9° of anterior slope in the right tibial plateau versus 6° posterior slope in the contralateral knee. The recurvatum was purely osseous. A diagnosis was made of proximal tibial anterior physeal premature closure leading to genu recurvatum.

**Fig. 2:**
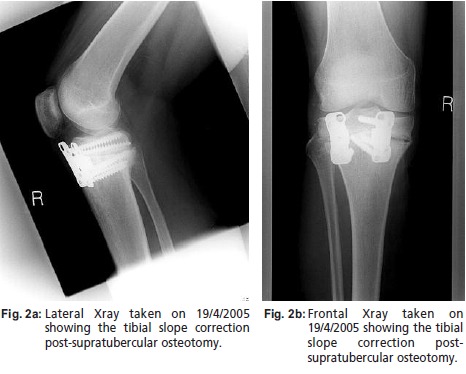
: (a)Lateral Xray taken on 19/4/2005 showing the tibial slope correction post-supratubercular osteotomy. (b) Frontal Xray taken on 19/4/2005 showing the tibial slope correction postsupratubercular osteotomy.

The patient was treated on 24th January 2005 with a 12.5 mm opening-wedge supra-tubercular proximal tibial osteotomy performed through a midline incision. The osteotomy was fixed with two anterior Puddu plates (Arthrex® Sheffield UK) and reinforced with bone substitute (HATriC™/ combining hydroxyapatite and tricalcium phosphate, Arthrex®|). A decision was made not to osteotomise the tibial tubercle as the patellar height was judged to be satisfactory. The follow-up (Fig. 2) was uneventful with a satisfactory clinical result. The implants were removed in 2006.

**Fig. 3:**
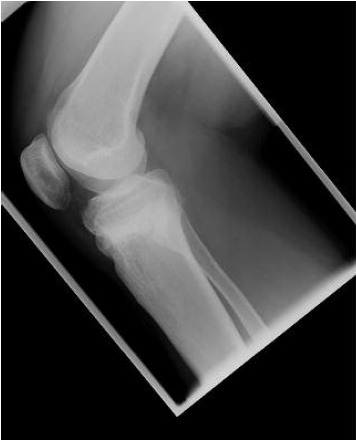
: Lateral Xray taken on 13/4/2011 following removal of the metalwork showing progression of the patella infera.

In 2011, the patient came back to clinic complaining of pain at the front of the right knee. The radiographs (Fig 3) revealed a patella infera (Caton-Deschamps index at 0.4, Insall-Salvati at 0.6, Blackburn-Peel at 0.5).

Table I shows a flowchart of the patient’s patellar height measurements

## Discussion

Proximal tibial growth arrest not due to a tibial traction pin has been described following femoral shaft fracture 1. It is probably due to trauma to the tibial tubercle and anterior proximal tibial physis at the time of injury^1^. The same forces that cause knee ligament injuries in adult femoral shaft fractures produce distal femoral or proximal tibial physeal injuries in the immature skeleton 2. The maturing physes of adolescents are particularly prone to injury, having the highest incidence of physeal injury from any cause; the proximal tibia being a common site.

The type V physeal injury described by Salter and Harris, after strong initial debate, is now widely accepted, although the mechanism of such injuries may be unclear (perhaps vascular rather than compression trauma). Salter and Harris postulated that type V fractures represented unrecognized compression injuries with normal initial radiographs that later produced premature physeal closure. The mechanism of the injury remains disputed with vascular deprivation rather than compression or crushing being postulated. Incidentally, the most common outcome of such an injury is closure of the tibial tubercle, often with the development of recurvatum deformity of the proximal tibia, after fractures of the femur or distal femoral epiphysis. Other locations and case reports of late physeal closure after extremity injury and apparently normal initial radiographs exist in the literature.(xx reference) This pattern of injury is unrecognized on initial radiographs. Undoubtedly, more sophisticated imaging of injured extremities (such as with MRI) will identify physeal injuries in the presence of normal plain radiograph.

Intramedullary internal fixation, including reamed rigid intramedullary nails, is the treatment of choice in adolescent femoral shaft fractures, even with open physes. To our knowledge, there is no mention in the literature of the nailing procedure in itself causing proximal tibial physeal growth arrest regardless of the nail entry point at the trochanter or piriform fossa.

For growth-arrest genu recurvatum in children with a deformity less than 20°, resection of the physeal bone bar alone has been advocated restorating 80% to 90% of growth^3^. In this case the patient was 17-years old when he came back to clinic with pain and hyperextension in his right knee. All long bone physes had already fused as per chronological age. The only option was an osteotomy. Internal fixation was chosen over progressive correction with a spatial frame 4 as it was a single-plane deformity with no associated shortening. Puddu plates are a recognized and popular fixation device for proximal tibial osteotomies.

Some evidence in the literature shows that for proximal tibial opening wedge osteotomies of up to 12.5 mm, bone grafting is not necessary at all. However, bone graft substitute provides added stability, osteoconduction and dead space obliteration, while obviating the need for autologous bone graft.

Anterior opening wedge osteotomy for genu recurvatum due to partial anterior proximal tibial premature physeal closure has already been described in the literature 3 but the risk of patella infera was not commented on. The recurvatum in our patient was still moderate compared to the case reported by Chen & al (2004). When the deformity reaches 15° there may be a functional complaint which worsens over time due to stretching of the posterior capsuloligamentous structures. This leads to impairment of the extensor mechanism resulting in weakness, instability and pain. Closing wedge anterior osteotomy or anterior displacement supra-tubercular osteotomy fixed with Steinmann pins have also been described for genu recurvatum in adolescents. It is, however, more invasive and technically more demanding, necessitating two incisions, extensive stripping of the proximal tibia, a fibular osteotomy (with the risk to the common peroneal nerve), and risk to the popliteal vessels when removing the base of the wedge.

In our case, the osteotomy was supra-tubercular, so the patella was inevitably brought down by the opening wedge. This could have been avoided by performing a tibial tubercle osteotomy and proximal transfer, or including the tibial tubercle as part of the osteotomy. Tibial tubercle osteotomy carries an extra risk of non-union and secondary displacement that has to be weighed against the potential benefits. The reported results for proximal tibial osteotomy distal to the tibial tubercle are poor. The correction of the deformity is less than good (probably because the site of the correction is too far from the site of the deformity). It leads to a prominent anterior curve of the tibial diaphysis. There has been no correlation between poor results and patella infera in supratubercular osteotomies in series reported. Nonetheless, anterior knee pain in the presence of patella infera is probably due to increased patellofemoral load following a change in contact areas.

Progressive patella infera is probably due to postoperative scarring of the patellar tendon 5. Table I shows that our patient had a rather low patella to start with, and that this worsened after the osteotomy.

**Table I:**
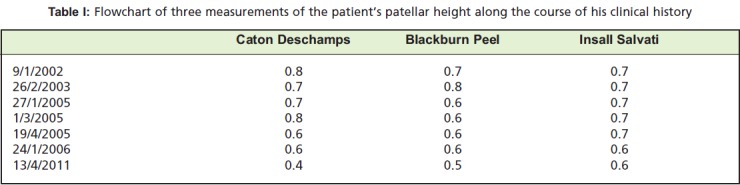
: Flowchart of three measurements of the patient’s patellar height along the course of his clinical history

## Conclusion

Femoral diaphyseal fracture in an adolescent with open physes may result in proximal tibial growth arrest. There is probably a role for MRI for early detection of such injury. For a single-plane genu recurvatum, high anterior opening wedge supra-tubercular osteotomy is a good option, with a risk of post-operative patella infera which may cause significant symptoms. At the time of the osteotomy, proximalisation of the tibial tubercle should be considered.
